# Inter- and intra-observer reliability of clinical movement-control tests for marines

**DOI:** 10.1186/1471-2474-13-263

**Published:** 2012-12-29

**Authors:** Andreas Monnier, Joachim Heuer, Kjell Norman, Björn O Äng

**Affiliations:** 1 Department of Neurobiology, Care Sciences and Society, Division of Physiotherapy, Karolinska Institutet, Huddinge, Sweden; 2 Swedish Armed Forces, Regional Medical Service Mälardalen, Berga, Sweden; 3 Fysiocenter Odenplan, Stockholm, Sweden; 4 Swedish Armed Forces, 1st Marine Regiment, 2nd Amphibious Battalion, Berga, Sweden

**Keywords:** Military, Motor control, Reproducibility, Screening, Sensitivity, Specificity, Validity

## Abstract

**Background:**

Musculoskeletal disorders particularly in the back and lower extremities are common among marines. Here, movement-control tests are considered clinically useful for screening and follow-up evaluation. However, few studies have addressed the reliability of clinical tests, and no such published data exists for marines. The present aim was therefore to determine the inter- and intra-observer reliability of clinically convenient tests emphasizing movement control of the back and hip among marines. A secondary aim was to investigate the sensitivity and specificity of these clinical tests for discriminating musculoskeletal pain disorders in this group of military personnel.

**Methods:**

This inter- and intra-observer reliability study used a test-retest approach with six standardized clinical tests focusing on movement control for back and hip. Thirty-three marines (age 28.7 yrs, SD 5.9) on active duty volunteered and were recruited. They followed an *in-vivo* observation test procedure that covered both low- and high-load (threshold) tasks relevant for marines on operational duty. Two independent observers simultaneously rated performance as “correct” or “incorrect” following a standardized assessment protocol. Re-testing followed 7–10 days thereafter. Reliability was analysed using kappa (κ) coefficients, while discriminative power of the best-fitting tests for back- and lower-extremity pain was assessed using a multiple-variable regression model.

**Results:**

Inter-observer reliability for the six tests was moderate to almost perfect with κ-coefficients ranging between 0.56-0.95. Three tests reached almost perfect inter-observer reliability with mean κ-coefficients > 0.81. However, intra-observer reliability was fair-to-moderate with mean κ-coefficients between 0.22-0.58. Three tests achieved moderate intra-observer reliability with κ-coefficients > 0.41. Combinations of one low- and one high-threshold test best discriminated prior back pain, but results were inconsistent for lower-extremity pain.

**Conclusions:**

Our results suggest that clinical tests of movement control of back and hip are reliable for use in screening protocols using several observers with marines. However, test-retest reproducibility was less accurate, which should be considered in follow-up evaluations. The results also indicate that combinations of low- and high-threshold tests have discriminative validity for prior back pain, but were inconclusive for lower-extremity pain.

## Background

Musculoskeletal disorders, especially in back and lower extremities, are common in marines [1,2] both during their basic military training [3] and later during service [4]. For marines, this could, as for many other military branches [5], reduce their operational efficiency and end their service prematurely [4,6-8]. Since it appears common that such problems lead to shift changes, the use of back-up personnel [5] or increased workloads for remaining personnel, one marine's disorders could affect the operational efficiency of an entire unit. Back and lower-extremity disorders have in addition been found to be a major contributor to reduction of marine unit strength before deployment, due to medical downgrading of the sufferers to non-deployment status [4]. Further, during deployment, musculoskeletal disorders are the most common causes of medical evacuation [9,10] and marines that suffer incidents of musculoskeletal disorder or spinal pain show less than 20% likelihood of returning to operational duty [10]. Early physical screening tests focusing on recruits' musculoskeletal health and function in relation to military duty are commonly used in modern armed forces. In the literature, however, no data on reliability exists on such clinical tests in marines.

Several studies in civilian populations have demonstrated a link between musculoskeletal disorders, pain and the ability to adequately control movements and muscular activation in clinical tests [11-13]. Some of these clinical tests are designed for focusing on movement control of a certain defined body region whilst actively moving an adjacent one. Such tests of movement control, also referred to in the literature as motor-control tests [14-16], low-load [15] or low-threshold [17] movement-control tests, are suggested for identifying deficits associated with repetitive low-load activity or static positioning [17]. It is suggested that such non-fatiguing movement-control tests will predominantly recruit slow motor units activated at a low threshold [17]. Clinical tests that on the other hand include high load or speed will involve recruitment of fast motor units [17], which are thus activated at a higher threshold and are less resistant to fatigue [18]. These high-threshold tests have therefore been suggested for identifying the risk of injuries in activities involving fatiguing or repeated high loads [17]. In our experience, based on clinical findings and empiric field observation with marines, tests covering low- and high-threshold movement control of the lower back and hip may adequately challenge weak-links in marines' musculoskeletal system as relevant in their operative duty. We believe such assessments to be suitable to include in protocols screening for deficits that may relate to musculoskeletal disorders, induced by exposures from various work tasks or postures in marines. Therefore, in this study, the tests included were selected to evaluate marines' ability to control or prevent defined movements of the lumbar spine and hip while performing specific lower extremity movement.

However, as the results of clinical testing may influence the testee′s future service or career, e.g. by possibly resulting in medical downgrading, it is of great importance for the tests to be reliable and valid for the specific group and its purpose. Specifically, since screening tests for military purposes are commonly used by multiple testers, good inter-observer reliability is required. If the test is to be used with follow-up evaluations, it needs also to show good intra-observer reproducibility [19]. Here, three important aspects influence measurement variability: variation related 1) to the observer(s), 2) to the instrument and the measuring procedure, and 3) to the subject tested [20].

Further, a clinically convenient test should show good validity, i.e. measure the entity that it purports to measure [20]. Specifically, evidence of discriminative validity is required to justify the use of clinical tests of musculoskeletal pain, i.e. how far the tests are able to differentiate between those with back- and lower-extremity pain and those without. Such testing accuracy may complement simple pain ratings, particularly in the work with early recognition of disorders, and possibly for planning further clinical examination and intervention. Useful clinical tests that aim to screen marines' physical function need, at the same time, to be simple and fairly brief since generally many personnel are being tested. In addition, methodological evaluations of clinical tests should advantageously be contingent on clinical contextual factors that reflect the testees' natural environment. Although clinical experience suggests that findings of impaired movement control relate to musculoskeletal disorders and pain episodes in the back and lower extremities, we have found no studies on movement-control tests that address such discriminative validity. Further, a few studies in civilian populations (subjects with back pain, subjects with musculoskeletal pain but not back pain and healthy controls) have evaluated the reliability of movement-control tests for the lower back and/or extremities, with inter-tester reliability ranging from poor to almost perfect/excellent [14,15,21-23], and intra-tester reliability from fair to excellent [21]. However, to our knowledge, there is no such published data on reliability of clinical tests in marines. The present aim was therefore to determine the inter- and intra-observer reliability of clinically convenient tests for assessing movement control of back and hip in marines. A secondary aim was to investigate the discriminative validity of the best fitting combination of tests for identifying back and lower-extremity pain disorders in this group of military personnel.

## Methods

### Study design

This inter- and intra-observer reliability study used a test-retest approach and *in-vivo* testing methodology. The study protocol included six standardized clinical tests that emphasize active movement control for back and hip. Performances were scored simultaneously by two, well-experienced physiotherapists (observers) who were familiar with the tests. The observers were blinded to each other's scores and to the subjects' health and background information. The procedure was repeated 7–10 (mean = 7.4) days thereafter. The six tests were to be assessed as ″correct″ (pass) or ″incorrect″ (fail), thus generating binominal data. Based on this, the sample size was calculated to approximately 34 subjects at a presumed agreement of 90% (CI 20%; chance agreement: 50%) [24] and enrolment was planned to meet this criterion. Written informed consent was obtained from all subjects, who received both written and oral information prior to participation. Confidentiality and voluntary participation were strongly stressed. The study was approved in advance by the Regional Medical Research Ethics Committee, Stockholm.

### Study sample

Thirty-three marines on active duty (assault infantry, combat craft crews and coastal rangers) were recruited from a combined company of the 2nd Amphibious Battalion, 1st Marine Regiment, Berga, Sweden, the main marine regiment in Sweden. Eligible subjects had to be in service during the test period. Excluded were subjects on limited duty due to illness (full- or part-time sick leave) and subjects temporarily posted or under training at the 2nd Amphibious Battalion. After receiving oral and written information, volunteering subjects were enrolled in the study and scheduled for testing. Of the 33 subjects enrolled, 32 were male and one female. Means (SD) for age, weight and height were: 28.7 (5.9) yrs, 82.5 (9.4) kg and 1.81 (0.059) m.

### Subjective measures of back and lower extremity pain

Standardized self-report questionnaires were used to collect demographic information and medical history for the previous six months, including numerical rating scales of ‘pain at present’ and for ‘the previous six months’ [25], specified by anatomical body region [26]. The numerical pain-rating scale has been found reliable and sensitive for the assessment of pain and has been suggested to be appropriate for use in clinical practice [25] and research [27]. For the purpose of this study, back- (lumbar, thoracic-back) and lower-extremity (hip/thigh, knee, ankle/foot regions) pain was defined as any reported pain experience (pain, ache or discomfort), and for pain at present this was ≥ 1 on the numerical pain-rating scale.

### Clinical tests

**Figure 1:**
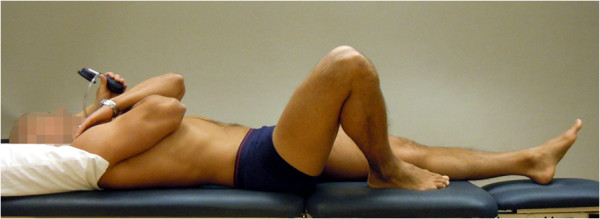
**The bent knee fall-out (BKFO) test (reproduced with permission from Movement Performance Solutions).** The BKFO test was used to test the ability to prevent rotation of the lumbar spine during abduction/lateral rotation (bent knee fall-out) of the hip. The test was classified as a low-load (threshold) test. Photos illustrates examples of views during the test. *Start position:* The subject lay supine with both legs straight. A pressure biofeedback unit (Chattanooga Group, Hixon, TN) was positioned between the lumbar lordosis and bench ipsilaterally to the test side and inflated to a pressure of 40 mmHg. A folded towel (towel thickness adjusted to match the height of the biofeedback unit) was placed between lumbar lordosis and bench on the contralateral side. The subject then flexed the hip and knee on the side to be tested until the foot was in line with the contralateral knee. Lumbar position was corrected (if needed) so that the biofeedback unit was 40 mmHg. *Test movement:* While preventing the lumbar spine from rotating, the subject lowered the bent leg out to the side, to 45° abduction/lateral rotation (bent knee fall-out) and then returned to the starting position. *Criteria:* Pass: ≤ 4 mmHg away from the initiating 40 mmHg during bent knee fall out.

**Figure 2:**
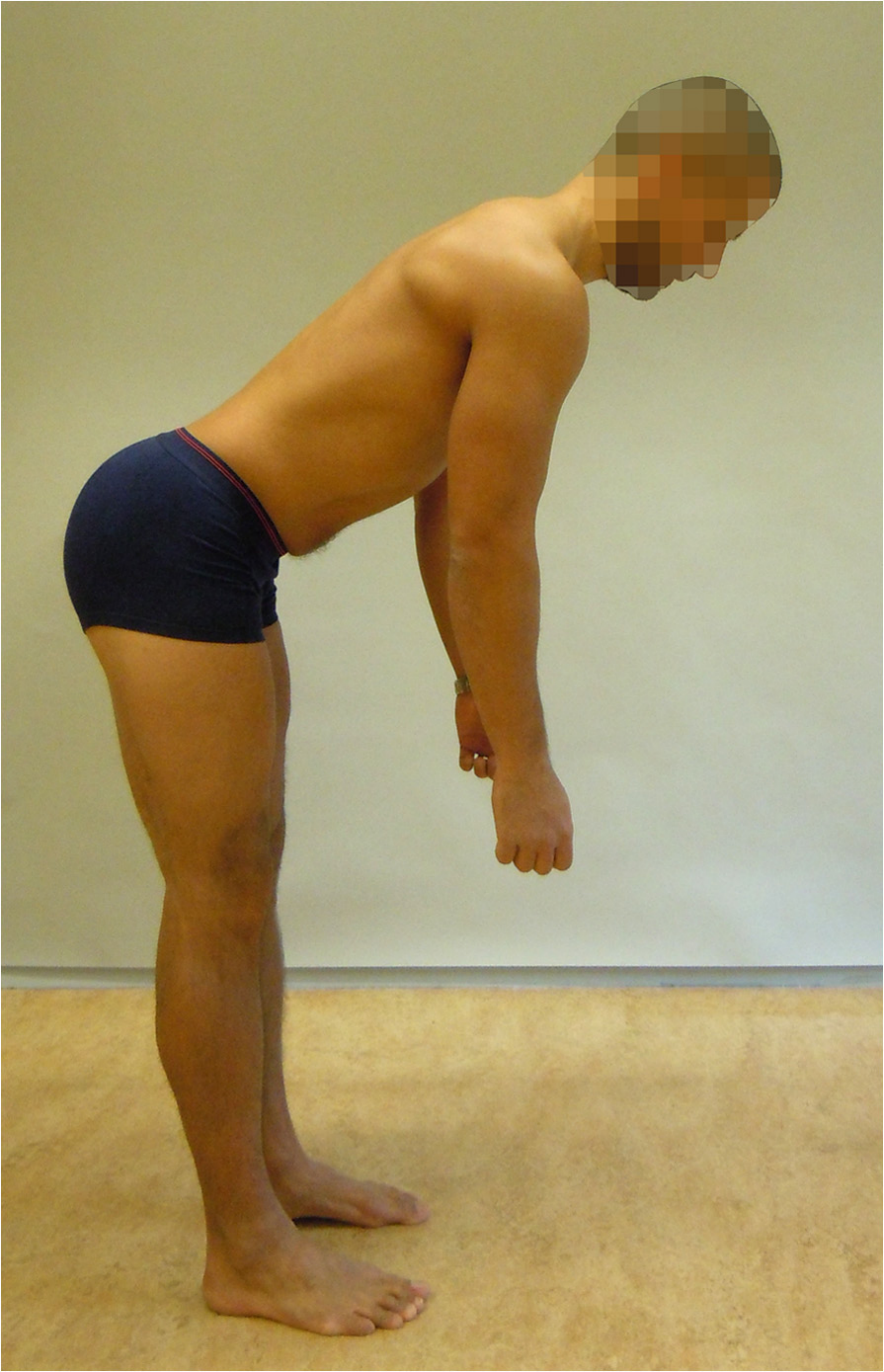
**The Standing bow (SB) test (reproduced with permission from Movement Performance Solutions).** The SB test was used to test the ability to prevent flexion of the lumbar spine during defined movement of the hip in upright standing. The test was classified as a low threshold test. Photos illustrates examples of views during the test. *Start position:* The subject stood with the pelvis relaxed and lumbar spine in neutral position. *Test movement:* While keeping the spine straight (not letting it round out or over arch) the subject bent the hips forwards to 50° (forward lean). *Criteria:* Pass: forward lean with maintenance of lumbar spine in a neutral position.

**Figure 3:**
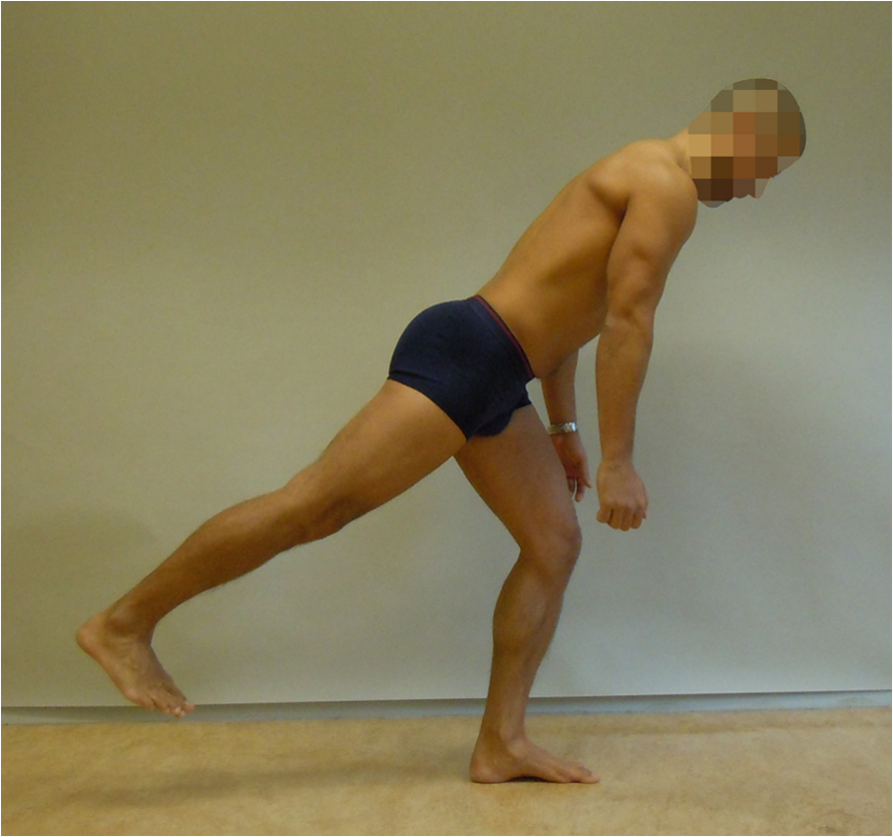
**The Single leg small knee bend+lunge-lean (SLKB+LL) test (reproduced with permission from Movement Performance Solutions).** The SLKB+LL test was used to test the ability to prevent motion of the lumbar spine and control movement of the hips during a lunge and forward-lean of the trunk, performed in a single leg knee-bend position. The test was classified as a low threshold test. Photos illustrates examples of views during the test. *Start position:* The subject stood with one foot back and the other in front (two feet lengths) from the rear foot. With the pelvis facing straight ahead and while keeping the back upright, the subject bent the knee to a forward lunge onto the front foot, allowing the rear heel to lift. *Test movement:* While keeping the spine straight, pelvis and chest facing ahead and knee and thigh over second toe, the subject bent forward at the hips to 45° forward leaning. The subject then lifted the rear foot off the floor and kept the leg extended, in a straight line with the body. This position was held for 5 s. *Criteria:* Pass: 5 s holding the position of 45° forward lean over the front foot with the rear leg extended in line with the trunk, while maintaining the lumbar spine in a neutral position and without changing pelvis or hip position.

**Figure 4:**
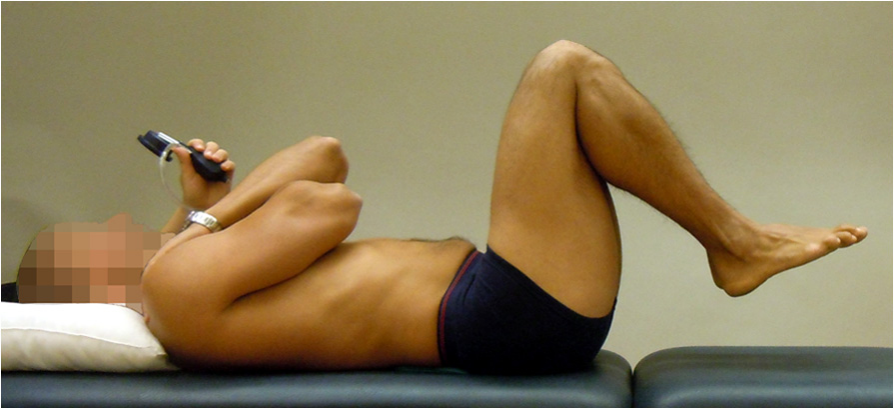
**The Double leg lift-lower (DLL-L) test (reproduced with permission from Movement Performance Solutions).** The DLL-L test was used to test the ability to prevent extension and flexion of the lumbar spine during defined movement of the hip, performed supine. The test was classified as a high threshold test. Photos illustrates examples of views during the test. *Start position:* The subject lay in crook lying (45° hip flexion), knees and feet together with arms folded across chest. A pressure biofeedback unit (Chattanooga Group, Hixon, TN) was positioned between the lumbar lordosis and bench and inflated to a pressure of 40 mmHg. *Test movement:* While preventing the lumbar spine from moving, the subject lifted both feet off the bench to 90° hip flexion and then returned to the starting position. *Criteria:* Pass: < 5 mmHg away from the initiating 40 mmHg while no movement in lumbar spine.

**Figure 5:**
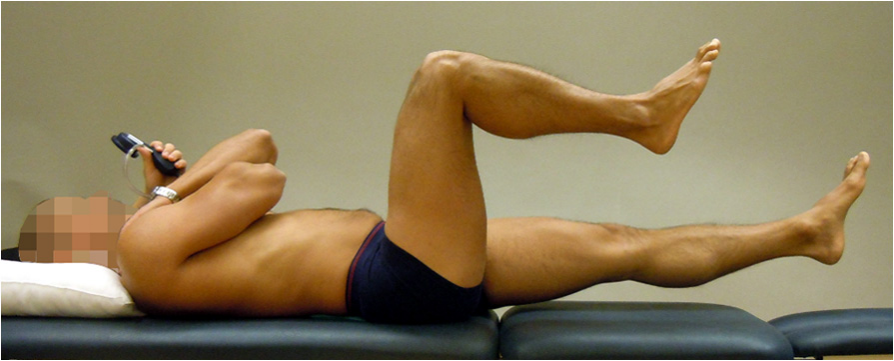
**The Double leg lift-alternate leg extension (DLL-ALE) test (reproduced with permission from Movement Performance Solutions).** The DLL-ALE test was used to test the ability to prevent extension, flexion and rotation of the lumbar spine, as well as leg abduction, lateral rotation and hip forward glide, during defined movement of the leg performed supine. The test was classified as a high threshold test. Photos illustrates examples of views during the test. *Start position:* The subject lay in crook lying (45° hip flexion), knees and feet together with the arms folded across the chest. A pressure biofeedback unit (Chattanooga Group, Hixon, TN) was positioned between the lumbar lordosis and bench, and inflated to a pressure of 40 mmHg. *Test movement:* While preventing the lumbar spine from moving (monitored with pressure biofeedback unit and visually), the subject lifted both feet off the bench to 90° hip flexion, then lowered and straightened one leg to fully extended position, and then back to 90° hip flexion. This movement was then repeated with the other leg, and both legs were then finally returned to starting position. *Criteria:* Pass: < 5 mmHg away from 40 mmHg while no movement in lumbar spine. The extending leg was not to move away from the midline or turnout.

**Figure 6:**
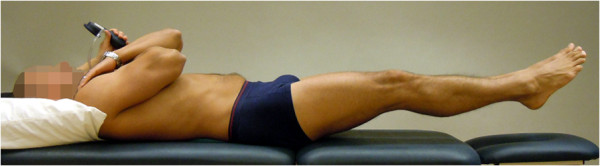
**The Double straight leg lower (DSLL) test (reproduced with permission from Movement Performance Solutions).** The DSLL test was used to test the ability to prevent extension and flexion of the lumbar spine during defined movement of the legs, performed supine. The test was classified as a high threshold test. Photos illustrates examples of views during the test. *Start position;* The subject lay in crook position (45° hip flexion), knees and feet together with arms folded across chest. A pressure biofeedback unit (Chattanooga Group, Hixon, TN) was positioned between the lumbar lordosis and bench and inflated to a pressure of 40 mmHg. *Test movement;* While preventing the lumbar spine from moving (monitored with pressure biofeedback unit) the subject lifted both feet off the bench to 90° hip flexion, then lowered and straightened both legs to fully extended, and then back to 90° hip flexion. *Criteria:* Pass: < 5 mmHg away from 40 mmHg while no movement in lumbar spine.

Six active movement-control tests were derived from descriptions by Comerford and Mottram [28,29]. These tests were Bent knee fall-out (BKFO; Figure 1), Standing bow (SB; Figure 2), Single leg small knee bend + lunge-lean (SLKB+LL; Figure 3), Double leg lift-lower (DLL-L; Figure 4), Double leg lift-alternate leg extension (DLL-ALE; Figure 5) and Double straight leg lower (DSLL; Figure 6). These tests, used in clinical treatment of the studied population, were initially selected based on discussions with clinicians with fairly long experiences of various clinical assessments in marines. Further, they are suggested for use with Swedish marines to screen and evaluate movement control of the back and hip. In principal, the tests were designed to evaluate the subjects' ability to control or prevent defined movements of the lumbar spine and hip, while performing specific movement in the lower extremity. Based on clinical/empirical knowledge with marines, we think that the loading complexity of the hip/lower back created by the active movement component in the tests adequately challenged field-observed weak links in marines' musculoskeletal system. For this reason these tests were performed in function-related positions/situations that adequately challenged these critical points of the musculoskeletal system. Three tests were classified as low-threshold (Figures 1, 2, 3) and three as high-threshold (Figures 4, 5, 6) [17], based on muscle activation levels/recruitment of slow- (low-threshold) and fast-twitch motor units (high-threshold) [30]. Two tests included movement control of both back and hip (Figures 3, 5) and four included movement control of the back (Figures 1, 2, 4, 6). Four tests were performed supine (Figures 1, 4, 5, 6) and two standing (Figures 2, 3). Initially a seventh test were included, ″plank and twist″ [28], but due to uncertainty over the rating criteria (not clearly observed during pre-study training) this test was early excluded. In four of the tests (Figures 1, 4, 5, 6) an air-filled pressure sensor (Pressure Biofeedback Unit, Chattanooga Group, Hixon, TN) was used to monitor lumbar movement. This sensor was originally developed for monitoring such spinal movement in clinical tests [31,32] and has proved useful for evaluating lumbar-muscle control [14-16] and neck-muscle function [33].

### Test protocol

After filling in initial questionnaires, each subject was instructed to wear only underwear so that movements of the lumbar spine, hips and lower extremities could be properly observed. The tests were performed in a standardized order (as specified from Figures 1, 2, 3, 4, 5, 6). The observers instructed every other enrolled subject as scheduled for test one (randomized for first subject), and this order was kept in the re-testing procedure. At first, the subjects received standardized instructions orally for the test and the instructing observer also demonstrated the test (see Additional file 1 for examples). All subjects then performed one trial with feedback and, if needed, received further visual, oral and/or manual instructions/guidance by the instructing observer. This was done in order to ensure full understanding of the test performance and to ensure that the test result did not reflect the subject's unfamiliarity with the movement. Subjects then performed the tests and the performance was assessed simultaneously by the two observers using a standardized assessment rating protocol (see Additional file 1 for details) and then dichotomized (i.e. ″fail″ if one direction in any region was uncontrolled). The test protocol took approximately 30 minutes to conduct. To familiarize themselves with the test procedure and protocol, the observers trained on *in vivo* observations on a total of nine subjects together, dispersed over two occasions before study start. Here, the observers discussed and synchronized test performance, instructions and rating procedure. During the testing, however, there was no such communication, and the observers were blinded to each other's scores.

### Analysis

Data on inter- and intra-observer agreement was determined by calculating kappa coefficients, 95% confidence intervals (CI) and percentage agreement. The kappa coefficient is the quantity of agreement, adjusted for chance [19,34]. It has a maximum of 1.0, indicating perfect agreement, while zero indicates agreement no better than chance [19]. When interpreting the strength of agreement for the kappa coefficient, we used the standards proposed by Landis and Koch [35], where a kappa coefficient was: 0 = poor, 0.01 – 0.20 = slight, 0.21– 0.40 = fair, 0.41– 0.60 = moderate, 0.61– 0.80 = substantial, and 0.81–1 = almost perfect. These standards were applied to the mean value from tests 1 and 2 (inter-observer reliability) and from test-re-test reproducibility (intra-observer reliability). To account for sampling error, the lower 95% CI of the obtained value of kappa coefficient should be > 0.2 [19]. The percentage agreement was calculated by dividing the numbers of agreed observations with the total number of observations for each test. The finite-population correction factor (fpc) [36] was applied to correct the variance on account of the sample size (n) in relation to the population (*N*):

$$\mathrm{fpc}=\left(\surd \left[\left(N–n\right)/\left(N–1\right)\right]\;\times \;\mathrm{SE}\right)$$

To investigate the discriminative validity of the best-fitting test/s for rated back- and lower-extremity pain during the previous six months and at present, respectively, the Akaike information criterion (AIC) auto-regression was used on initial data (test 1). Hence, the lower the AIC value generated, the better the fit of the model:

AIC=2k–2L,

where k is the number of variables (or tests) in the statistical model and L is the log likelihood of the model. A regression model with more than three variables/tests or a sensitivity less than 60% test was considered to be of limited value. A subject with both back- and lower-extremity pain was analysed in both groups. To check for systematic differences between tests one and two (systematic bias), McNemar analyses were applied with a p value of 0.05 as significant.

## Results

One subject was lost to follow-up and one subject elected not to complete the follow up DSLL test due to recurrent back pain. Twenty-three subjects (70%) rated that they had had back and/or lower extremity pain the previous six months, and eleven subjects (33%) rated that they had ongoing back and/or lower extremity pain (see Table 1 for details).

**Table 1:** Rated back and lower-extremity (LE) pain the previous six months and at present (n = 33)

**Ratings**	**Pain previous six months**			**Pain at present**	
		**Worst pain**^**a**^	**Average pain**^**a**^		**Pain at present**^**a**^
	**n**^**c**^**(%)**	**md (range)**	**md (range)**	**n**^**c**^**(%)**	**md (range)**
No pain	10 (30)			22 (67)	
Back pain^b^	18 (55)			9 (27)	
Thoracic pain	12 (36)	5 (1–7)	2 (1−7)	5 (15)	1 (1–5)
Lumbar pain	15 (45)	3 (2–10)	1 (1−6)	7 (21)	2 (1–6)
LE pain^b^	16 (48)			8 (24)	
Hip/Thigh pain	2 (6)	2 (2–2)	1 (1−1)	0 (0)	0 (0)
Knee pain	14 (42)	3 (1–8)	2 (1−5)	6 (18)	3 (1–4)
Foot/ankle pain	5 (15)	7 (2–8)	3 (1−5)	3 (9)	3 (2–7)
Only Back pain	7 (21)			3 (9)	
Only LE pain	5 (15)			2 (6)	
Back and LE pain^b^	11 (33)			6 (18)	

^a^Pain rated on numeric rating scale, NRS.

^b^Subjects could rate pain in more than one region.

^c^Number of subjects that rated ≥1 on numeric pain ratings the previous six months and at present, respectively.

### Inter-observer reliability

Table 2 shows kappa coefficients (κ) and 95% confidence intervals representing inter- observer reliability. Three tests, BKFO (κ_test 1_= 0.89; κ_test 2_ = 1.00), DLL-L (κ_test 1_ = 0.87; κ_test 2_ = 0.93) and DLL-ALE (κ_test 1_ = 0.84; κ_test 2_ = 0.87), reached almost- perfect inter-observer reliability with mean κ-coefficients from tests 1 and 2 > 0.86. Results for two of the remaining tests, SLKB+LL (κ_test 1_ = 0.60; κ_test 2_ = 0.63) and DSLL (κ_test 1_ = 0.53; κ_test 2_ = 0.81), showed substantial inter-observer reliability with mean κ-coefficients > 0.61, while for SB (κ_test 1_ = 0.49; κ_test 2_ = 0.63) the inter-observer reliability was moderate with mean κ-coefficients of 0.56. A lower value of the 95% CI > 0.2, representing both first and second testing, was noted for all tests except DSLL and SLKB+LL.

**Table 2:** Inter-observer reliability: kappa coefficient, 95% confidence intervals, percent agreement and standard error

**Test**	**BKFO**	**SB**	**SLKB+LL**	**DLL-L**	**DLL-ALE**	**DSLL**
Test 1						
Kappa coefficient (CI 95%)	0.89 (0.70-1.00)	0.49 (0.21-0.77)	0.60 (0.27-0.92)	0.87 (0.63-1.00)	0.84 (0.63-1.00)	0.53 (0.16-0.90)
% agreement	97.0	75.8	87.8	90.9	93.9	87.9
Std error	0.107	0.153	0.180	0.101	0.111	0.203
Test 2						
Kappa coefficient (CI 95%)	1 (1.00-1.00)	0.63 (0.39-0.87)	0.63 (0.19-1.00)	0.93 (0.80-1.00)	0.87 (0.72-1.00)	0.81 (0.58-1.00)
% agreement	100.0	81.3	93.8	96.9	93.8	93.6
Std error	0	0.132	0.240	0.070	0.086	0.126
Mean kappa coefficient^a^	0.95	0.56	0.61	0.87	0.86	0.67

Abbreviations: BKFO, Bent knee fall-out; SB, Standing bow; SLKB+LL, Single leg small knee bend + lunge-lean; DLL-L Double leg lift-lower; DLL-ALE, Double leg lift-alternate leg extension; DSLL, Double straight leg lower; CI 95%, 95% confidence intervals; Std error, standard error.

^a^Mean value of kappa coefficient obtained from test 1 and test 2.

### Intra-observer reliability

Table 3 presents kappa coefficients and 95% confidence intervals for intra-observer reliability. Three tests, BKFO (κ_obs. A_ = 0.64; κ_obs. B_ = 0.53), SB (κ_obs. A_ = 0.48; κ_obs. B_ = 0.39), and DLL-L (κ_obs. A_ = 0.63; κ_obs. B_ = 0.40) had moderate intra-observer reliability with mean κ-coefficients of 0.44-0.58. The remaining three tests, SLKB+LL (κ_obs. A_ = 0.43; κ_obs. B_ = 0.31), DLL-ALE (κ_obs. A_ = 0.39; κ_obs. B_ = 0.21), and DSLL (κ_obs. A_ = 0.24; κ_obs. B_ = 0.32) had fair intra-observer reliability with mean κ-coefficients of 0.22-0.37. For observer A, lower 95% CI exceeded 0.2 in three tests (BKFO, SB and DLL-L), while observer B ended with a lower 95% CI of less than 0.2 for all tests. The McNemar analysis showed that significantly more subjects passed the DLL-ALE test on re-testing than at the initial testing procedure (Table 4). This was significant for both observers (p = 0.026 and p = 0.003). For observer B, this was also significant (p = 0.026) for the DLL-L test.

**Table 3:** Intra-observer reliability: Kappa coefficient, 95% confidence intervals, percent agreement and standard error

**Test**	**BKFO**	**SB**	**SLKB+LL**	**DLL-L**	**DLL-ALE**	**DSLL**
Observer A						
Kappa coefficient (CI 95%)	0.64 (0.21-1.00)	0.48 (0.20-0.77)	0.43 (0.01-0.86)	0.63 (0.40-0.86)	0.39 (0.14-0.65)	0.24 (0–0.61)
% agreement	93.8	75.0	87.5	81.3	68.8	78.1
Std error	0.232	0.156	0.232	0.126	0.140	0.203
Observer B						
Kappa coefficient (CI 95%)	0.53 (0.11-0.95)	0.39 (0.10-0.67)	0.31 (0–0.68)	0.40 (0.15-0.66)	0.21 (0–0.43)	0.32 (0–0.69)
% agreement	90.6	68.8	81.3	68.8	57.6	78.1
Std error	0.228	0.157	0.203	0.138	0.120	0.202
Mean kappa coefficient ^a^	0.58	0.44	0.37	0.52	0.30	0.22

Abbreviations: BKFO, Bent knee fall-out; SB, Standing bow; SLKB+LL, Single leg small knee bend + lunge-lean; DLL-L Double leg lift-lower; DLL-ALE, Double leg lift-alternate leg extension; DSLL, Double straight leg lower; CI 95%, 95% confidence intervals; Std error, standard error.

^a^ Mean value of kappa coefficient obtained from observer A and B.

**Table 4:** Test results presented for each observer/test and number of cases identified that failed the test

	**Test 1**	**Test 2**	***Number of cases identified at test one with pain, that failed the test***	
	**Pass/fail**	**Pass/fail**		
			**Back pain prev. 6 mo/at present**	**LE pain prev. 6 mo/at present**
Observer A				
BKFO	28/5	30/2	1/1	4/1
SB	12/21	14/18	12/7	9/6
SLKB+LL	5/28	3/29	17/9	14/8
DLL-L	15/18	21/11	9/4	7/3
DLL-ALE	9/24	17/15	14/6	11/7
DSLL	4/29	7/24	18/9	14/8
Observer B				
BKFO	27/6	30/2	1/1	4/1
SB	14/19	18/14	11/7	8/6
SLKB+LL	7/26	3/29	15/8	14/7
DLL-L	14/19	22/10	11/6	7/5
DLL-ALE	7/26	19/13	15/7	12/8
DSLL	6/27	7/24	16/7	13/8

Abbreviations: LE, lower extremity; BKFO, Bent knee fall-out; SB, Standing bow; SLKB+LL, Single leg small knee bend + lunge-lean; DLL-L Double leg lift-lower; DLL-ALE, Double leg lift -alternate leg extension; DSLL, Double straight leg lower.

### Sensitivity/specificity

Table 5 shows a discriminative summary of the best-fitting test(s) for back and lower-extremity pain rated for the previous six months and at present, respectively. For observer A, the best fitting tests were BKFO and DSLL (AIC = 41.8, 94%/47% (sens/spec)) for back pain the previous six months while, for observer B, a five-variable model (AIC = 42.9, 89%/67% (sens/spec)) including BKFO and DSLL emerged as best fitting tests for back pain the previous six months. However, when including only BKFO and DSLL for observer B, sensitivity/specificity were reduced by six and seven percent, respectively (AIC = 44.2, 83%/60% (sens/spec)). For both observers, the BKFO and DSLL model discriminated prior back pain if the BKFO test was passed and the DSLL test failed. Regarding lower-extremity pain, results for observer A revealed that the best-fitting tests for pain the previous six months were DLL-L and BKFO (AIC = 45.2, 81%/59% (sens/spec)). Here, the model discriminated prior lower extremity pain not only in cases failing both tests, but also in those passing both tests. However, for observer B, DLL-L and SLKB+LL (AIC = 45.7, 44%/88% (sens/spec)) best predicted lower-extremity pain the previous six months, i.e. when passing DLL-L and failing SLKB+LL. A combination of low- and high-threshold tests consistently emerged as having the best fit. However, no model was sensitive for discriminating back and lower-extremity pain at present (sensitivity <60%) (Table 5).

**Table 5:** **Discriminative analysis: Akaike information criterion (AIC), *p*-value and sensitivity/specificity of model variables for pain ratings**

	**Model**					**Discriminative analysis**		
	**Var. 1**	**Var. 2**	**Var. 3**	**Var. 4**	**Var. 5**	**AIC**^**a**^	***p *value**	**Sens/spec (%)**
Back pain								
prev. 6 month								
Observer A	BKFO	DSLL				41.84	0.008	94/47
Observer B	BKFO	SB	SLKB+LL	DSLL	DLL-ALE	42.89	0.012	89/67
at present								
Observer A	SB	SLKB+LL	DLL-L			36.68	0.046	33/92
Observer B	SB					40.49	0.140	0/100
LE pain								
prev. 6 month								
Observer A	BKFO	DLL-L				45.24	0.040	81/59
Observer B	SLKB+LL	DLL-L				45.73	0.050	44/88
at present								
Observer A	SLKB+LL	DLL-L	DLL-ALE			36.10	0.038	50/92
Observer B	DLL-ALE					36.10	0.034	0/100

Abbreviations: Sens, sensitivity; spec, specificity; LE, lower extremity; BKFO, Bent knee fall out; SB, Standing bow; SLKB+LL, Single leg small knee bend + lunge-lean; DLL-L Double leg lift-lower; DLL-ALE, Double leg lift -alternate leg extension; DSLL, Double straight leg lower.

^a^ AIC, Akaike information criterion = 2k – 2L (k = number of variables; L = log likelihood).

## Discussion

We sought to determine the inter- and intra-observer reliability of six clinical tests targeted for screening and following marines' ability to perform accurate movement control. The tests had moderate-to–almost-perfect inter-observer reliability while intra observer reliability was fair-to-moderate. Discriminative regression revealed that combinations of low- and high-threshold tests had discriminative validity for previous back pain, but were inconclusive for lower-extremity pain.

Since the recruited marines were on active duty, and not recruited from subjects seeking care, the external validity extends only to a population of marines on active service. This was felt to be a strength of the study since the selected tests were intended and limited for this operational group. The results could however be of interest for researchers and clinicians alike, particularly for those working with similar military units. Further, we used an *in-vivo* study procedure similar to our clinical or preventive work in respect of settings and rating criteria, hence strengthening the ecological validity of the study protocol. Here, also, a large number of military personnel are usually tested and screened in a short time frame, and we therefore applied one practice round for each test. Some of our tests included sub-scores on observations and ratings for several body regions (SLKB+LL and DLL-ALE) and more than one direction of movement (SLKB+LL, DLL-L, DLL-ALE and DSLL). We believe, however, that when applying clinical tests for screening purposes, for logistical reasons an overall screening examination should be used, for example, to set priorities for further individual clinical action, but also to collect data for epidemiological analyses and follow-up. Notably, the procedure with two observers scoring the same subject simultaneously, here with one observer instructing the subject, limits the inter-observer reliability to test performance only. Considering our discriminative regression, the number of subjects was rather small (n = 33), and this was why we pooled lumbar and thoracic pain as back pain, and hip/thigh, knee, ankle/foot pain as lower-extremity pain, respectively, in this analysis. Further, for defining pain at present, a cut-off of ≥1 NRS may seem low. However, our experience with marines is that they underestimate their level of pain, also learned in other groups [37], and therefore a cut-off point of 1, which equals ″any pain experience″, was selected. In addition, a study on US Army soldiers [37] showed that the prevalence of back pain history may be underestimated in long-term recall surveys compared to monthly follow-ups. Developing a standardized operational definition to determine functional limitation, including pain ratings and pain interference with activity (operational efficiency), may improve the reliability of a future outcome construct in marines, thus possible improving its potential on discriminative and predictive validity.

Our data on inter-observer reliability ranged from moderate to almost perfect agreement (Table 2). While no such reliability data exist on movement control tests in marines, our results on agreement between observers are consistent with [14,15,22], or somewhat better than [21,23], most other reliability studies of movement control conducted in the civilian population. Here, Enoch et al. [14] and Roussel et al. [15] presented good (moderate-to-excellent) inter-observer reliability with their *in-vivo* collected data, though few tests were similar to ours in terms of test protocol (c.f. BKFO and SB). We believe, however, that our results indicate that the present six clinical tests are reliable for use in screening programs with multiple observers in marines.

Our results on intra-observer reliability were fair-to-moderate. Surprisingly few studies report on the intra-observer reproducibility of movement control tests, particularly since such clinical tests are commonly used for follow-up evaluation. The results of Luomajoki et al. [21] ranged from fair to excellent intra-observer reliability for ten movement-control tests. Two of our tests (c.f. BKFO and SB) were similar to theirs, though our corresponding kappa coefficients indicated lower reproducibility than theirs. However, their test-retest ratings were based on video recordings of one test occasion.

Interestingly, for two of the tests in the present study, i.e. DLL-L and DL-ALE, more subjects ″passed″ the re-test procedure than on the initial test occasion (Table 4). Such results may reflect a learning effect of the tests themselves (or systematic bias), and can only be manifested using observation from repeated testing. This probably also applies to our lower kappa coefficients on these tests. There were no clear indicators for any specific test being more difficult to instruct or evaluate relating it to poor re-test reproducibility. Further, only one other study [14] of movement control tests discloses how many practice times the subjects were allowed for each test. However, their study design did not include test-retest measurements, thus no intra-reliability analyses. Even so, we believe that repeated practice rounds may reduce learning effect, thus influence test reproducibility positively. In addition, improvement on the repeated test emphasizes the importance of including within-subject variation in test-re-test data relevant for clinical interpretation. Future studies, however, need to consider a trade-off between ″realistic″ amount of practice rounds related to their clinical work and sufficient elimination of learning effects.

One of the tests, the SLKB+LL, showed substantial inter-observer reliability at the re-test, with a kappa coefficient of 0.63, but with a percentage agreement as high as 94%. This discrepancy was probably due to an uneven number that passed/failed the test (Table 4), and it demonstrates how the kappa coefficient could be affected by such prevalence [38]. In order to adjust prevalence effects on kappa values, different types of adjustment have been discussed [19]. For example, with the prevalence-adjusted bias-adjusted kappa (PABAK), the adjusted kappa may be calculated with a maintained level of agreement, hence creating a ″hypothetical population″ with optimal distribution of pass/fail ratio [39]. Such adjusted coefficients may indeed add to the understanding of external validity extended to other populations such as other military units or possibly in civilian contexts. However, prevalence effects on kappa coefficients are themselves informative in a particular population [40] and, within the present study aim, we elected to report conventional kappa only. Further, the SLKB+LL and the DSLL showed lower 95% CIs of the kappa coefficient of less than 0.2 on one test occasion, respectively, thus indicating an increased risk of measurement error. For intra- observer reliability, this was also so for most of the tests for both observers, here probably affected by the present learning effects. This should be considered in follow-up evaluation and interpretation with the present clinical tests.

Regarding discriminative validity, our results indicate that combinations of low- and high- threshold movement-control tests had some discriminative validity for previous back pain, but not for present pain. Concerning lower extremity pain, there were differences in sensitivity/specificity between observer A and B, also for tests included in best fitting model, thus limiting the discriminative power of these observations. While we have learned that the AIC auto-regression rather accurately separates tests that do not really relate/contain properties with the dependent variable, pre-selection of tests with good kappa-coefficients may have strengthened our regression model. However, we believe our discriminative findings are an important complement to pain ratings, particularly since altered motor control may persist after pain relief [11] and long-term recall of pain may be underestimated [37], as indicated above. Our results somewhat support the use and interpretation of test combinations, rather than information from single tests. Since the BKFO and DSLL model discriminated prior back pain if the BKFO (low-threshold test) was passed, and the DSLL (high-threshold test) failed, the clinical and physiological implication of such results should be further validated. Even so, this is interesting since Roussel et al. [15] showed that two movement-control tests could predict injury in the back or lower-extremity over six months in professional ballet dancers. However, within the limits of the present study, the direction of causality is uncertain. In other words, does the pain experience cause certain results with movement control or vice versa. Also, our results say little about future incidents, and we believe therefore that further research should address the predictive validity of movement-control tests for musculoskeletal disorders in marines. Such knowledge would certainly contribute to the evidence for use of such screening tests in this group of military personnel.

## Conclusions

Clinical tests that emphasize movement control for back and hip had moderate-to-almost-perfect inter-observer reliability, indicating that these tests are reliable as screening tests using several observers with marines. However, test-retest reproducibility was not as accurate, with intra-observer reliability ranging from fair to moderate. This should be considered in follow-up evaluation. Our results also indicated that combinations of low- and high-threshold movement-control tests had discriminative validity for earlier back pain, but were inconclusive for lower-extremity pain. Further studies should emphasize predictive validity with clinically convenient tests for musculoskeletal disorders among marines.

## Competing interests

The authors declare that they have no competing interests.

## Authors’ contributions

AM participated in the conception and design of the study, acquisition, analysis and interpretation of the data and was the main writer of the paper. JH participated in the planning, acquisition and interpretation of the data as well as writing and revising the paper. KN was involved in the design of the study, acquisition of the data and revising the paper. BOÄ was the senior project researcher, participating in the conception and design of the study, analyzing and interpreting the data, and writing and revising the paper. All the authors read and approved the final manuscript.

## Pre-publication history

The pre-publication history for this paper can be accessed here:

http://www.biomedcentral.com/1471-2474/13/263/prepub

## Supplementary Material

Additional file 1 **Appendix A.** (Inter-and intra-observer reliability of clinical movement-control test for marines). Click here for file
