# Supplementary material for: Inter- and intra-observer reliability of clinical movement-control tests for marines
Source: BMC Musculoskelet Disord. 2012 Dec 29;13:263. doi: 10.1186/1471-2474-13-263 (PMC3552977; doi:10.1186/1471-2474-13-263)
Supplement: Additional file 1 — Appendix A. (Inter-and intra-observer reliability of clinical movement-control test for marines). [file 1471-2474-13-263-S1.doc]

| ***Single Leg Small Knee Bend + Lunge & Lean + Heel Lift*** | |
| --- | --- |
| ***Start***  ***Position*** | o Stand with one foot forward and one foot back  o Front foot is 2 foot lengths in front of rear foot  o Inside edge of the front foot aligned straight ahead  o Keeping heel down, bend the knee to lunge forward onto the front foot allowing the rear heel to lift. (The kneecap should be vertically aligned above the toes)  o Keep the thigh out over the second toe  o The back should be upright (vertical as if sliding down a wall) (Small Knee Bend position)  o The pelvis should be facing straight ahead (not rotated away from the front foot) |
| ***Test***  ***Movement*** | • **Bend forward at the hips to 45° forward leaning while keeping the**  **spine straight (don’t let it round out or over arch)**  • **Keep the pelvis and chest facing straight ahead**  • **Keep the knee and thigh over the second toe**  • **With the front foot flat, lift the rear toe just clear of the**  **floor keeping the rear leg extended in a straight line with the**  **body**  • **There should be a straight line from the point of the shoulder**  **through the trunk and down the rear leg (angled at about 45°)**  • **Do not bend too far on the front hip (trunk not horizontal)**  • **Hold the position for 5 seconds** |
| ***Benchmark*** | 45° forward lean over the front foot with the rear leg extended in line with the body |

| ***Observation Criteria*** |  | |
| --- | --- | --- |
| **Low Back/Pelvis** | **Left** | **Right** |
| Can you prevent the back from rounding out (flexing)? | No ❑ | |
| Can you prevent the back from over arching (extending)? | No ❑ | |
| Can you prevent rotation of the pelvis? (pelvis stays facing straight ahead) | No ❑ | No ❑ |
| Can you prevent side bending of the pelvis/trunk? | No ❑ | No ❑ |
| **Hip** | | |
| Can you prevent the front (WB) hip from flexing past 450 as the rear leg lifts? (i.e. trunk drops forwards towards horizontal) | No ❑ | No ❑ |
| Can you prevent turning in of the weight-bearing (WB) front knee? | No ❑ | No ❑ |
| Can you prevent the pelvis side shifting laterally on the supporting (WB) leg as you lift the rear leg? | No ❑ | No ❑ |
| Can you prevent the (NWB) rear leg dropping into flexion from the straight line? | No ❑ | No ❑ |
| Can you prevent abduction away from the midline or turnout (lateral rotation) of the (NWB) rear leg as it extends behind you? | No ❑ | No ❑ |

| ***Analysis for this Study*** |  |
| --- | --- |
| For this study the above information was dichotomised to "correct” (pass) or “incorrect” (fail) if one box (or more) were crossed | ***Results*** |
|  | **Fail** =  |
| **Single leg small knee bend + lunge & lean** | ❑ |

| ***Double Leg Lift & Alternate Leg Extension*** | |
| --- | --- |
| ***Start***  ***Position*** | Lie on the back, legs bent with knees and feet together and arms folded across your chest  o Place a Pressure Biofeedback Unit (PBU) under the centre of the lumbar lordosis with the pelvis relaxed and neutral  o Inflate the PBU to a base pressure of 40 mmHg  o Monitor the position of the pelvis with visual observation and with the PBU |
| ***Test***  ***Movement*** | **• Keeping the pelvis from moving, slowly lift both feet (at the same time) off the floor until the thighs are vertical (hip flexion to 90°)**  **• Lower one foot back towards the floor**  **• Keeping that foot just above the floor, slowly straighten that leg to the fully extended (horizontal) position and then lower the heel to touch the floor**  **• Slide the foot back towards the hip along the floor and then back so that the thighs are vertical**  **• The low back/pelvis should not move into rotation, arch into**  **extension or flatten into flexion**  **• Now repeat on the other side**  **• After performing the test on both sides, lower both feet (together) to the floor**  **• Try to keep the PBU pressure at 40 mmHg.**  **• A pressure change (increase or decrease) of greater than 4 mmHg (2 graduations) indicates uncontrolled movement. A change of up to 4 mmHg is acceptable but 5 mmHg or more is not**  **• A pressure change of less than 5 mmHg is acceptable while the legs are moving, only if the trunk can be re-stabilised at 40 mmHg when the legs stop moving at the heel touch point** |
| ***Benchmark*** | Double leg flexion to 90° and full single leg extension + double leg lower |

| ***Observation Criteria*** |  | |
| --- | --- | --- |
| **Low Back/Pelvis** | **Left** | **Right** |
| Can you prevent low back from flattening and rounding during the test movement (prevent an increase of pressure by 5 mmHg or more) ? | No ❑ | |
| Can you prevent low back from arching during the test movement? (prevent a decrease of pressure by 5 mmHg or more) | No ❑ | |
| Can you prevent the pelvis from rotating? (observe for pelvic rotation) | No ❑ | No ❑ |
| **Hip** | | |
| Can you prevent the extending leg from moving away from the middline or turning out? | No ❑ | No ❑ |
| Can you prevent a ‘clunk’ (forward slipping of the head of the hip joint) as the non weight-bearing (NWB) leg extends? | No ❑ | No ❑ |

| ***Analysis for this Study*** |  |
| --- | --- |
| For this study the above information was dichotomised to "correct” (pass) or “incorrect” (fail) if one box (or more) were crossed | ***Results*** |
|  | **Fail** =  |
| **Double Leg Lift & Alternate Leg Extension** | ❑ |

| ***Double Straight Leg Lower*** | |
| --- | --- |
| ***Start***  ***Position*** | Lie on the back, legs bent with knees and feet together and arms folded across your chest  o Place a Pressure Biofeedback Unit (PBU) under the centre of the lumbar lordosis with the pelvis relaxed and neutral  o Inflate the PBU to a base pressure of 40 mmHg  o Demonstrate that tilting the pelvis forwards and arching the back results in a decrease in pressure (extension) and demonstrate that rolling the pelvis backwards and rounding the back results in an increase in pressure (flexion)  o Monitor the position of the pelvis with visual observation and with the PBU |
| ***Test***  ***Movement*** | •  **Keeping the pelvis from moving slowly lift both feet (at the same time) off the floor until the thighs are vertical (hip flexion to 90°)**  • **Slowly lower both feet back towards the floor and once the feet are just off the floor start to extend both legs out**  • **Keeping the feet just above the floor, continue straightening the legs until the knees and hips are fully extended**  •  **With the legs fully extended, lower both heels to touch the floor**  •  **The low back/pelvis should not arch into extension or flatten into flexion**  •  **Return to the start position keeping the PBU pressure at 40 mmHg.**  •  **A pressure increase of 5 mmHg or more (45 or above) =**  **uncontrolled flexion**  •  **A pressure decrease of 5 mmHg or more (35 or below) =**  **uncontrolled extension**  •  **A pressure change of less than 5 mmHg is acceptable while the legs are moving, only if the trunk can be re-stabilised at 40 mmHg when the legs stop moving at the heel touch point** |
| ***Benchmark*** | Full double leg extension + lower to double heel touch |

| ***Observation Criteria*** |  | |
| --- | --- | --- |
| **Low Back/Pelvis** | **Left** | **Right** |
| Can you prevent low back from flattening and rounding during the test movement (prevent an increase of pressure by 5 mmHg or more)? | No ❑ | |
| Can you prevent low back from arching during the test movement? (prevent a decrease of pressure by 5 mmHg or more)? | No ❑ | |

| ***Analysis for this Study*** |  |
| --- | --- |
| For this study the above information was dichotomised to "correct” (pass) or “incorrect” (fail) if one box (or more) were crossed | ***Results*** |
|  | **Fail** =  |
| **Double Straight Leg Lower** | ❑ |
